# Peanut Shell Derived Carbon Combined with Nano Cobalt: An Effective Flame Retardant for Epoxy Resin

**DOI:** 10.3390/molecules26216662

**Published:** 2021-11-03

**Authors:** Jing Liang, Wenhao Yang, Anthony Chun Yin Yuen, Hu Long, Shuilai Qiu, Ivan Miguel De Cachinho Cordeiro, Wei Wang, Timothy Bo Yuan Chen, Yuan Hu, Guan Heng Yeoh

**Affiliations:** 1 School of Mechanical and Manufacturing Engineering, University of New South Wales, Sydney, NSW 2052, Australia; jing.liang1@unsw.edu.au (J.L.); i.decachinhocordeiro@unsw.edu.au (I.M.D.C.C.); wei.wang15@unsw.edu.au (W.W.); timothy.chen@unsw.edu.au (T.B.Y.C.); g.yeoh@unsw.edu.au (G.H.Y.); 2 State Key Laboratory of Fire Science, University of Science and Technology of China, 96 Jinzhai Road, Hefei 230026, China; yangwenhao@mail.ustc.edu.cn (W.Y.); yuanhu@ustc.edu.cn (Y.H.); 3 School of Mechanical Science and Engineering, Huazhong University of Science and Technology, Wuhan 430074, China; longgan88@hust.edu.cn

**Keywords:** peanut shell, physical barrier effect, carbon, catalysis effect, flame retardant

## Abstract

Biomass-derived carbon has been recognised as a green, economic and promising flame retardant (FR) for polymer matrix. In this paper, it is considered that the two-dimensional (2D) structure of carbonised peanut shells (PS) can lead to a physical barrier effect on polymers. The carbonised sample was prepared by the three facile methods, and firstly adopted as flame retardants for epoxy resin. The results of thermal gravimetric analysis (TGA) and cone calorimeter tests indicate that the carbon combined with nano Cobalt provides the most outstanding thermal stability in the current study. With 3 wt.% addition of the FR, both peak heat release rate (pHRR) and peak smoke production rate (PSPR) decrease by 37.9% and 33.3%, correspondingly. The flame retardancy mechanisms of the FR are further explored by XPS and TG-FTIR. The effectiveness of carbonised PS can be mainly attributed to the physical barrier effect derived by PS’s 2D structure and the catalysis effect from Cobalt, which contribute to form a dense char layer.

## 1. Introduction

Epoxy resin (EP) is one of most important thermosetting polymers. It is used for surface coating, adhesive, paint, and insulating materials for electrical devices due to its high toughness, low curing shrinkage, outstanding mechanical properties, excellent adhesion, perfect solvent and chemical resistance [1,2,3]. Nevertheless, EP is highly flammable and is involved with toxic volatiles during combustion (i.e., CO). Moreover, the massive emission of aromatic compounds and hydrocarbons also results in producing smoke, which seriously threatens the safety of the public during fire scenarios and limits its versatility [4].

Various flame retardants (FRs) have been developed to reduce the flammability of EP. Halogen-based agents are one of the most effective FRs due to their gaseous phase mechanisms [5]. However, owing to the emission of a high volume smoke and corrosive gas (i.e., HBr or HCl), traditional halogen-based FR agents have been gradually replaced by halogen-free nanoscale FRs, which contain compounds such as phosphorus, silicon, nitrogen or minerals [6,7]. Owing to the large interfacial area between polymers and nano-fillers, relatively low filler loading leads to a significant reduction in peak heat release rate (PHRR), and also superior mechanical and physical properties. Among inorganic nanomaterials, two-dimensional nanoscale fillers such as graphene and its derivatives [8,9], Layered Double Hydroxides (LDH) [10,11], Montmorillonite (MMT) [12] and Hexagonal Boron Nitride (h-BN) [13,14], have displayed outstanding fire retardancy performance due to the ultra-high aspect ratio and physical barrier effect [15,16].

With the consciousness of the environment and human health, the development of biomass-based FR additives has been increasingly encouraged and explored. In recent years, research interests have been attracted to biomass-derived carbon. It is a low-cost, carbon-rich material sourced from the biomass. After the annealing treatment, the stable carbon backbone leads to acceptable thermal stability. The porous structure with a high surface area can cause pore infiltration by polymer, enhancing the mechanical properties [17,18]. Numerous studies have reported the effectiveness of using biochar-based wood and agriculture waste as fillers on different polymers. Sabzoi et al. added carbonised rice husk into polylactide (PLA) and it was discovered that the addition of biochar in PLA promoted its thermal and mechanical properties [19]. Samuele et al. prepared biochar derived from softwood, rice husk and oilseed rape as FRs for ethylene-vinyl acetate (EVA). The results indicated that increasing the loadings (15, 20, 40 wt.%) of biochar could improve the fire behaviour of EVA by significantly decreasing PHRR and total heat release [20]. Liang et al. synthesised a flame retardant agent based on phosphate and urea-grafted bamboo charcoal for polylactic acid (PLA), and the results presented that it could improve flame retardancy of PLA significantly. With 30 wt.% addition of the filler, The LOI reached 32.1 vol% and passed the UL-94 V-0 grade vertical flame test [21]. However, very limited studies have reported about biochar from peanut shells.

There have been some recent studies about the application of carbon derived from peanut shells in the energy storage system [20,21,22]. Jia et al. [23] firstly reported their work about a hybrid sodium ion capacitor (NIC) being derived from peanut shells. Huanlei et al. [24] improved the electrochemical performance of the sodium-ion battery by using hierarchical porous carbon derived from peanut skin as an anode. In both of their work, the sheet-like morphology of biochar was observed. Therefore, it is expected that peanut shell-derived carbon with a 2D structure can improve the flame retardant performance of EP due to its stable thermal stability and physical barrier effect.

In this paper, peanut skin was carbonised as the only raw material at different temperatures in the first two methods. On the other hand, considering the catalysis effect of transition metal [25,26,27], Cobalt [28,29] was chosen to combine with carbonised peanut shells to improve the charring formation in the third method. The main objectives of the reported work herein are summarised as follows:

(1) Developing three kinds of flame retardants based on carbon derived from peanut shells to improve the flame performance of EP through facile ways;
(2) Evaluating the effectiveness of neat EP and its composites by conducting characterisation and benchmark fire safety tests such as cone calorimeter;
(3) Analysing the influence of carbonisation degree and the incorporation of nano Cobalt on fire safety behaviour of EP;
(4) Investigating flame retardant mechanisms from two aspects including condensed phase and gaseous phase with the help of material characterisation techniques.

## 2. Materials and Methods

### 2.1. Raw Materials

In this study, peanut shells (PS) were purchased from the local market and the wasted skin was collected as raw material for biochar preparation. Co(NO_3_)_2_ 6H_2_O and concentrated sulfuric acid (98%) was purchased from Sigma Aldrich (Shanghai, China). Epoxy resin (Bisphenol-A type) and curing agent (diaminodiphenylmethane) were purchased from Shixian Chemical Industry Co., Ltd. (Nanjing, China) and Sinopharm Chemical Reagent Co., Ltd.(Shanghai, China), respectively. 

### 2.2. Preparation

**Scheme 1:**
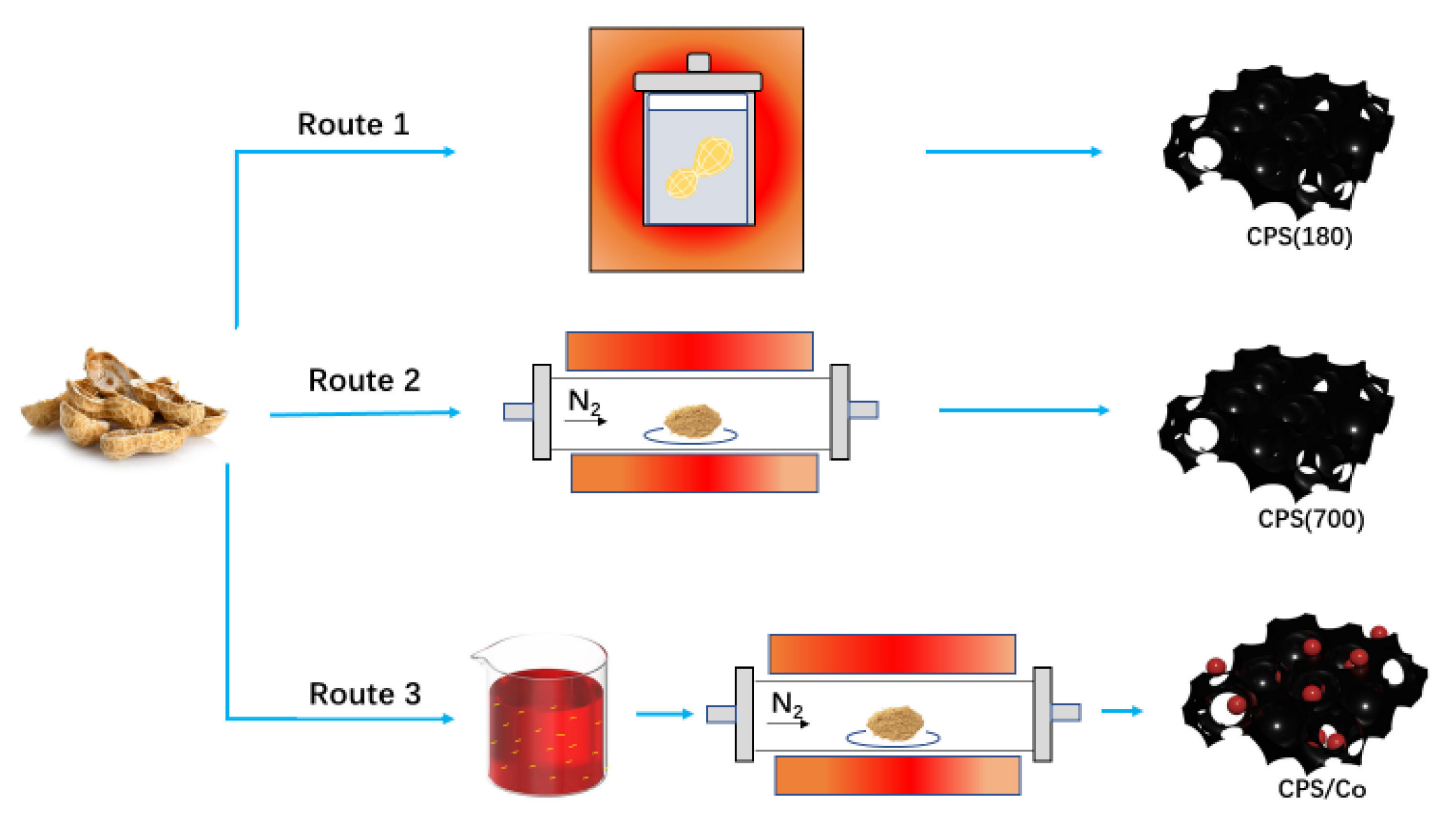
Three routes for fabrication process of flame retardants.

To prepare, raw peanut shell skin was dipped in Deionized (DI) water for 24 h, then dried at 60 °C for 24 h. After that, the peanut shells were ground by using PQ-N04 Gear Drive 0.4-Liter Planetary Ball Mill (Nanda instrument Co., Nanjing, China) with 600 rpm speed for 30 min. Then, flame retardants based on peanut shells (PS) were prepared by three simple routes. The fabrication process is shown in Scheme 1. Route one is the hydrothermal method mentioned in [24]. The mixture of 15.0 g peanut skin powder and 25 mL diluted sulfuric acid were enclosed in a 500 mL Teflon-lined stainless-steel autoclave. The autoclave was maintained at 180 °C for 48 h and then cooled down naturally. The product was collected by filtration, washing with deionized (DI) water for 5 times, drying at 60 °C for 24 h, and then labelled as CPS (180). In route two, 15.0 g peanut shell skin powder was placed in a tubular furnace at 700 °C (with a heating rate of 5 °C/min) for 3 h under argon flow. After activation treatment, the product was collected and labelled as CPS (700). For route three, 58.3 g Co (NO_3_)_2_ · 6H_2_O was completely dissolved in 200 mL of deionised water. Then 15.0 g peanut shell powder was dipped in the prepared Co (NO_3_)_2_ solution for 24 h. Afterwards, the powder was collected by filtration, drying at 60 °C for 24 h, and then placed in tubular furnace at 700 °C (with heating rate of 5 °C/min) for 3 h under argon flow. The resulting product was collected and labelled as CPS/Co.

A typical synthesis process of EP composite with 3 wt.% addition of flame retardant agent was as the following steps. Firstly, a homogeneous suspension was prepared by mixing 1.0 g of FR and 100 mL acetone with ultrasonication. Then 27 g of EP thick liquid was added into the suspension with constant mechanical stirring speed 1000 rpm for 24 h. After acetone was removed from the mixture by heating at 80 °C for 3 h, 6 g of pre-melting curing agent was slowly poured into the EP/FR suspension and stirred for 3 min. Finally, the mixture was poured into the module and cured at 100 °C and 150 °C for 2 h, marking as EP, and composites EP/CPS (170), EP/CPS (700) and EP/CPS/Co, respectively.

### 2.3. Measurements

X-ray diffraction (XRD) measurements were conducted for the structure analysis by using an X-ray diffractometer (Rigaku Co., Tokyo, Japan). The microscopic features of flame retardants based on peanut shell-derived carbon, coated with a gold layer in advance, were observed by scanning electron microscopy (TM4000Plus, Hitachi Co., Tokyo, Japan). Carbonisation degree of flame retardants (carbons derived from peanut shells) was recorded by the Raman spectra at ambient temperature by a LabAM-HR Raman spectrometer (Jobin Yvon Co., Paris, Ltd., France) with a 532 nm argon laser. Fire performance was accessed by using a cone calorimeter (Fire Testing Technology, West Sussex, UK) according to ISO 5660 standard procedures, using 100 × 100 × 30 mm^3^ specimens at an external heat flux of 35 kW/m^2^. Thermal gravimetric analysis (TGA) was performed on a TGA, NETZSCH TG209 F3 Tarsus by heating from 50 °C to 800 °C at a heating rate of 20 °C min^−1^. Thermogravimetric analysis/infrared spectrometry (TG-IR) was conducted to analyse the gaseous emission during TGA test by combining a TGA Q5000 thermogravimetric analyser and a Nicolet 6700 FT-IR spectrophotometer. X-ray photoelectron spectroscopy (XPS) was used to investigate char residue after cone calorimeter by VG ESCALAB MK-II electron spectrometer (V.G. Scientific Ltd., London, UK).

## 3. Results and Discussion

### 3.1. Characterisation of Flame Retardants

**Figure 1:**
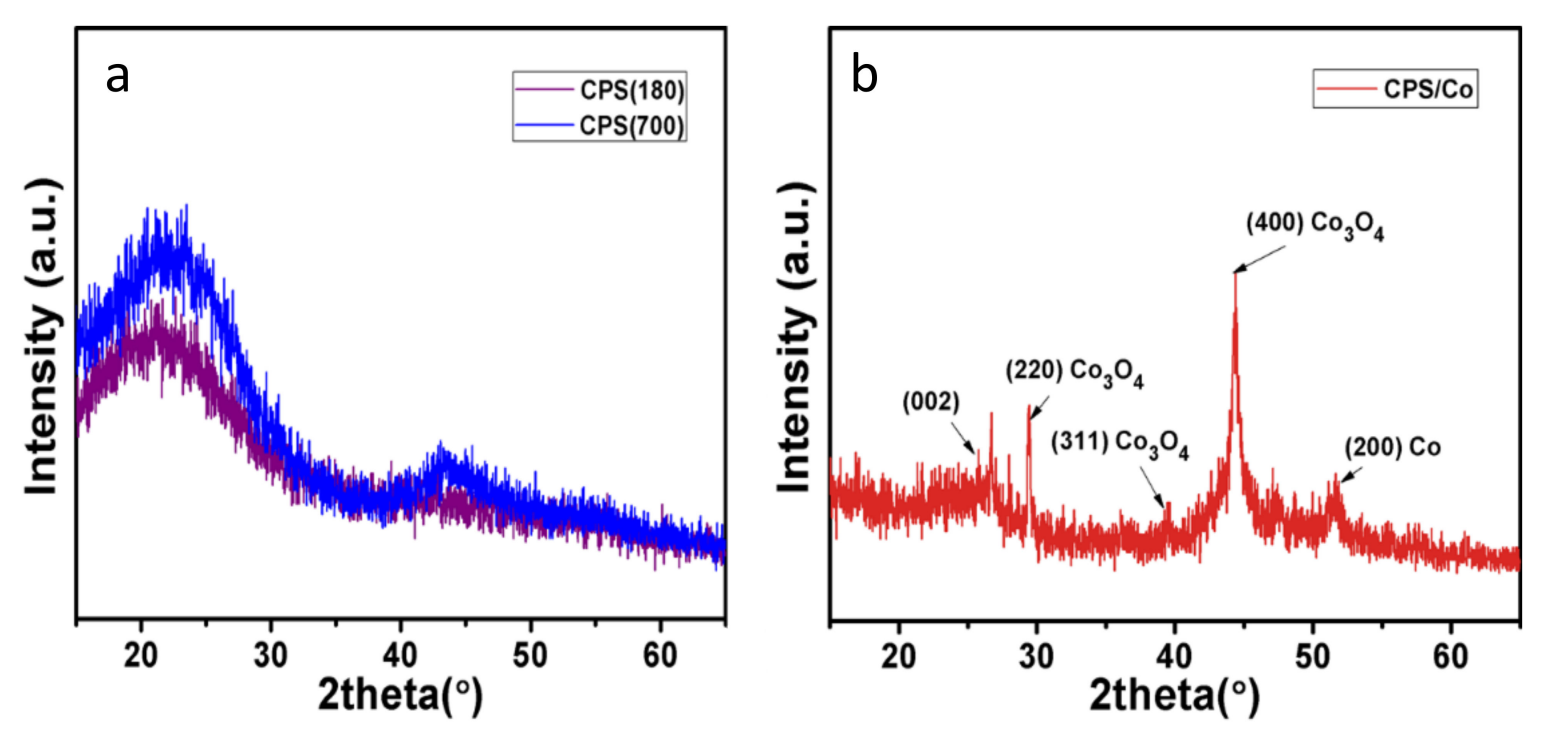
XRD patterns of FR: (**a**) CPS (180) and CPS (700), (**b**) CPS/Co.

Figure 1 shows the XRD curves of CPS (180), CPS (700) and CPS/Co. It can be observed that for both CPS (180) and CPS (700) board diffraction peak appears at around 21, which is close to graphite (002) [30]. It is attributed to typical amorphous carbon, indicating the formation of a graphite structure to some extent. For CPS/Co, in addition to the broad diffraction peak (002) [31], the XRD spectra shows strong diffraction peaks at 29.6, 39.7 and 44.4, corresponding to the characteristic crystal planes of Co_3_O_4_ (220) (311) and (400) [32,33]. Moreover, the diffraction peak at 51.6 is related to the characteristic crystal plane (200) of Co element [34].

**Figure 2:**
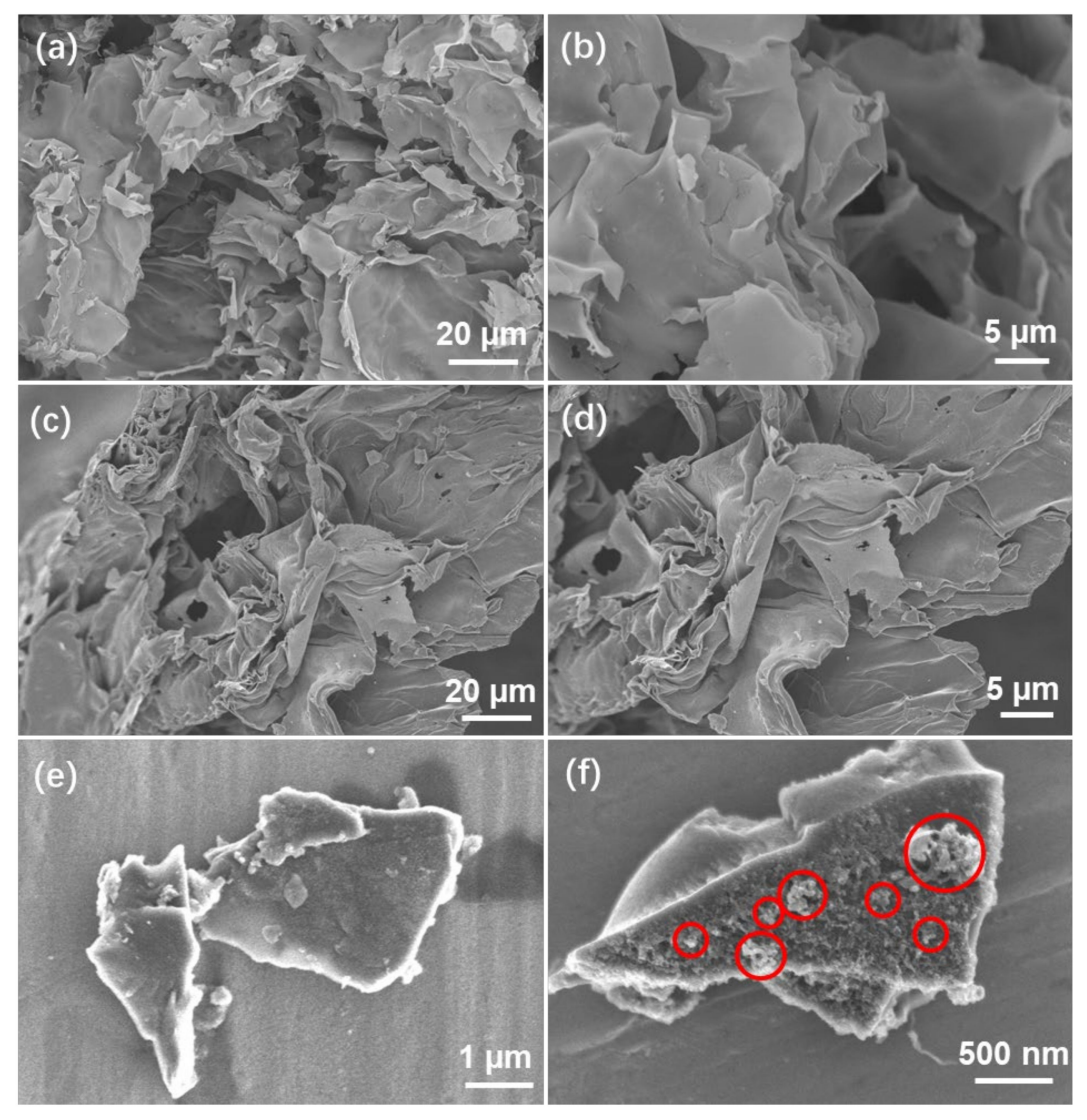
SEM images of prepared carbon derived from peanut shells:(**a**,**b**) CPS (180) before grinding, (**c**,**d**) CPS (700) before grinding, (**e**) CPS (700) after grinding, (**f**) CPS/Co (Cobalt oxide particles marked by red circles).

The morphology of CPS (180), CPS (700) and CPS/Co was investigated by SEM. To show layer porous structures clearly, samples for CPS (180) and CPS (700) before grinding are displayed in Figure 2a–d. Figure 2e exhibits the sample for CPS (700) after grinding, which was found to own a sheet-like 2D structure. When combing with Co, Cobalt oxide particles (marked by red circles) can be observed to grow on top of carbon derived from PS in Figure 2f. The SEM images indicate that with a layered structure, the FR filler based on carbonised PS can form a 2D structure after grinding. With acceptable thermal stability, this 2D carbon can benefit fire performance due to the physical barrier effect, similar to the two-dimensional nanoscale fillers mentioned in the introduction. Scanning electron microscopy images and corresponding elemental mapping images of CPS/Co are provided in Figure S1 in the Supplementary Materials. Moreover, Figure S2 in Supplementary display SEM images of cross-sections, suggesting well dispersed FRs in the polymer matrix.

**Figure 3:**
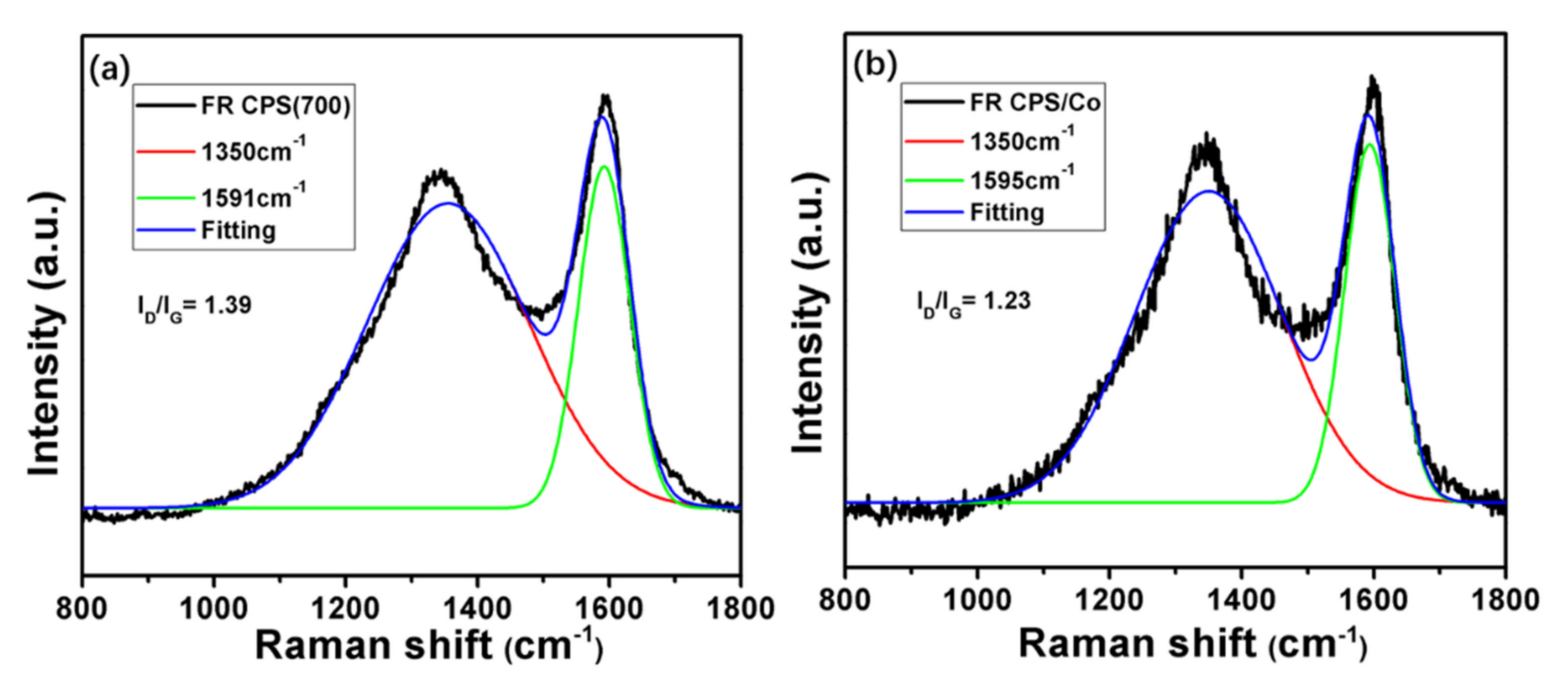
Raman spectra of the flame retardants: (**a**) CPS (700) (**b**) CPS/C.

Raman spectroscopy was employed to quantify the graphitisation degree of the carbons derived from the peanut shells. The Raman spectra of CPS (180) is provided in Figure S2 in the Supplementary Materials. The only board band peaking at around 1590 cm^−1^ for CPS (180) infers the formation of amorphous carbon [35,36], while the spectra of CPS (700) and CPS/Co in Figure 3 display two peaks at approximately 1350 cm^−1^ and 1590 cm^−1^, corresponding to D and G bands, respectively. These two peaks indicate the graphite-like structure and a higher graphitisation degree. The area ratio of D to G band (I_D_/I_G_) is used to assess the quality of carbons [37,38]. The lower I_D_/I_G_ value also suggests a higher carbonisation degree [39]. The value ratio of D to G band for CPS (700) is 1.39, whereas the value is around 1.23 for CPS/Co, suggesting that CPS/Co has the highest carbonisation degree. Considering CPS (700) and CPS/Co were prepared at the same temperature (700 °C), it is evident that transition metal Co improves the quality of carbon derived from peanut shells during the preparation process.

### 3.2. Thermal Stability of EP and Its Composites

**Figure 4:**
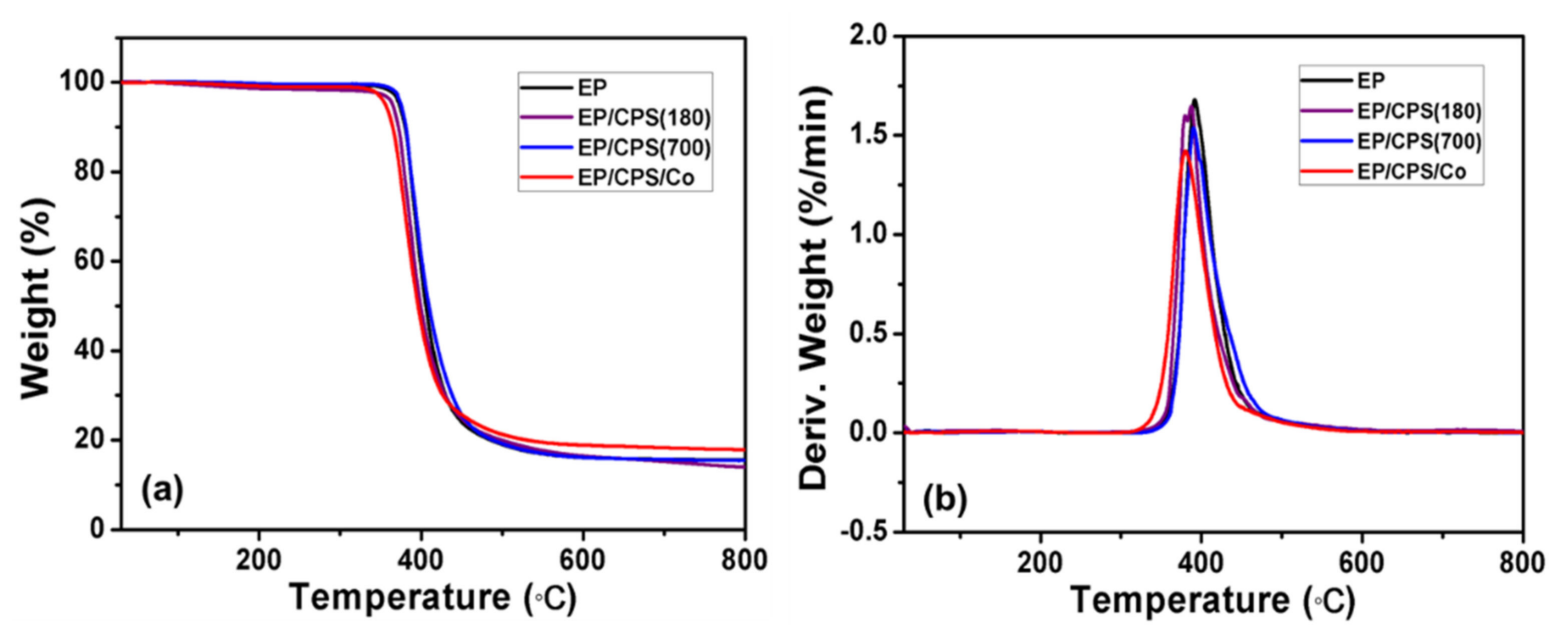
(**a**) TG and (**b**) DTG curves for EP, EP/CPS (180), EP/CPS (700) and EP/CPS/Co in nitrogen atmosphere at a heating rate of 20 °C/min.

Thermal gravimetric analysis (TGA) was also conducted to evaluate the thermal stability and degradation process of EP and other composites. Figure 4 displays the (a) TGA and (b) DTG curves of all samples under nitrogen, and the corresponding detail data are summarised in Table 1. Similar thermal degradation behaviours can be observed for all the samples in Figure 4a. The one-stage decomposition process is mainly ascribed to the break of the macromolecular chains [40].

T_5%_ is defined as the temperature at 5% mass loss, commonly treated as the initiation of the decomposition [38,41]. T_max_ denotes the temperature at the maximum mass loss rate. Compared to EP, the values obtained for EP/CPS (180) in T_5%_, T_max_ and char residue at 700 °C decreased slightly due to the unstable amorphous carbon structure. While for EP/CPS (700), although there are no substantial changes for T_max_ and char residue, T_5%_ increases a little, indicating a delay in degradation. At the same time, comparing with neat EP, the maximum mass loss rate of EP/CPS (700) decreases from 1.68 to 1.54%/min, which is due to the acceptable thermal stability of CPS (700) at a lower temperature (under 400 °C) [42].

On the other hand, the incorporation with Co oxide leads to a drop in T_5%_ and T_max_, which can be ascribed to the catalysis effect from Co_3_O_4_ and boost the thermal degradation of composite EP/CPS/Co. Nevertheless, compared to neat EP, the char residue of EP/CPS/Co rises from 15.69 to 18.31%. Furthermore, in Figure 4b the value of maximum mass loss rate of EP/CPS/Co is lowest among all the samples, inferring that CPS/Co can restrain mass loss during the decomposition process and display the significant thermal stability in the stage of decomposition above 400 °C.

### 3.3. Fire Performance of EP and Its Composites

It is widely known that Cone calorimeter is considered as a useful instrument to assess the force burning fire performance of materials. Some key parameters such as peak heat release rate (pHRR), total heat release (THR), peak smoke production rate (PSPR) and total smoke release (TSR) of each specimen were measured and listed in Table 2. Additionally, compared to neat EP, percentage reductions of pHRR, THR, PSPR and TSR for all samples, were calculated and listed in Table 3.

**Figure 5:**
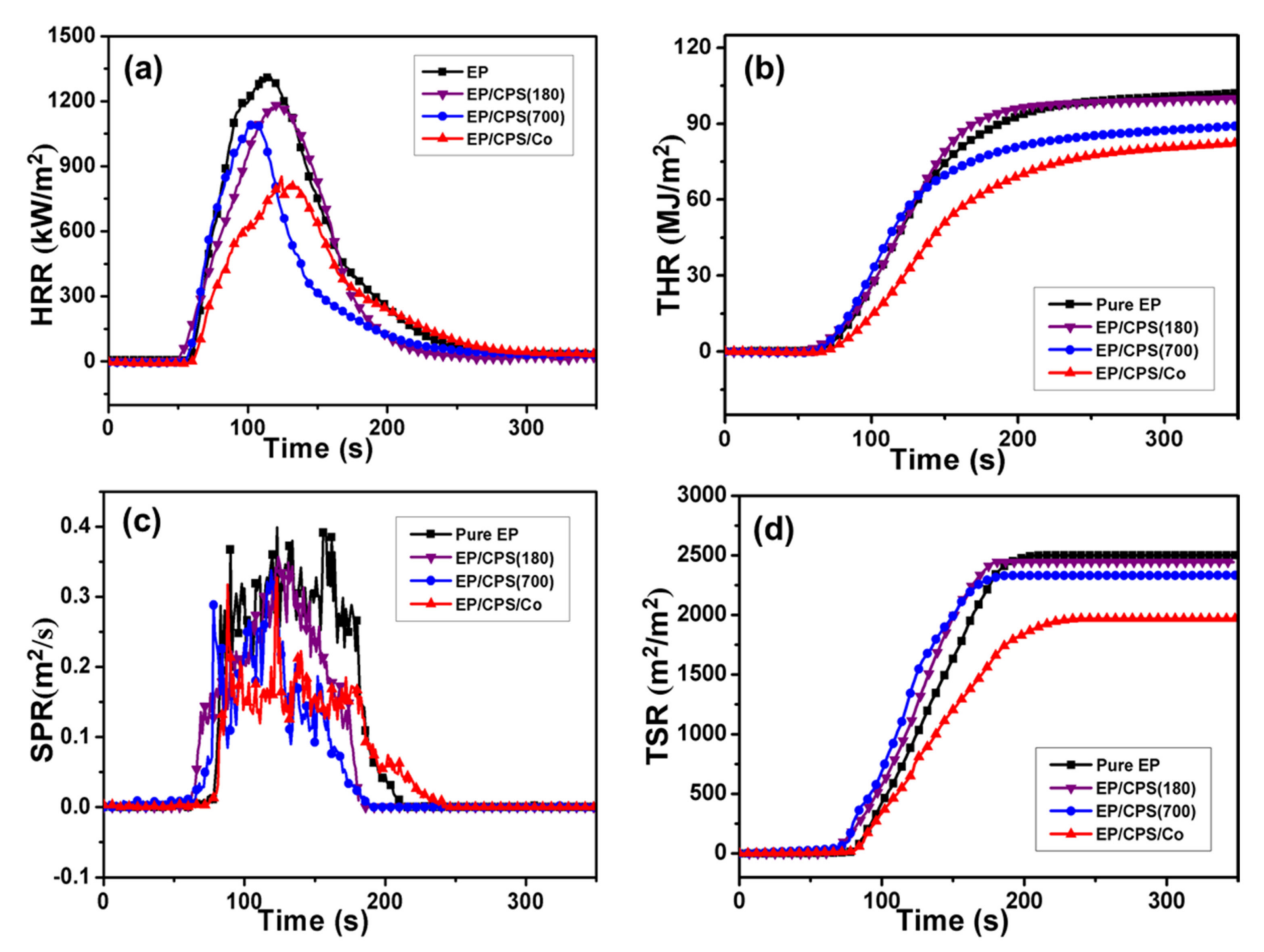
(**a**) Heat release rate, (**b**) total heat release, (**c**) smoke production rate, (**d**) total smoke release for EP and EP composites.

The introduction of all FRs additives brought no obvious changes for the time to ignition (TTI). The HRR and THR curves of EP and its composites are shown in Figure 5a,b. Neat EP burns rapidly with high pHRR and THR, with a value of 1313 kw/m^2^ and 101 MJ/m^2^, respectively. Although the loading of CPS (180) does not deliver any significant improvement, the flame retardancy of EP has been improved by adding CPS (700) and CPS/Co. Specifically, compared with neat EP, the pHRR of EP/CPS (700) decreased by 17.4%, from 1313 kw/m^2^ to 1084 kw/m^2^. The value of THR was also reduced by 12.6%, from 102 MJ/m^2^ to 89 MJ/m^2^. Meanwhile, the pHRR of EP/CPS/Co dropped by 37.9%, from 1313 kw/m^2^ to 816 kw/m^2^. Additionally, the THR value decreased by 19.3%, from 102 MJ/m^2^ to 82 MJ/m^2^. Therefore, the addition of CPS/Co leads to the most efficient reduction of pHRR and THR. Similarly, the lowest value of MARHE (maximum average rate of heat emission), which represents the mean HRR curve to some extent, was noted for EP/CPS/Co (426.0 kw/m^2^).

Figure 5c,d displays SPR and TSR vs. time curves. Neat EP releases a large amount of smoke during burning, which is extremely harmful for human health. As expected, the loading of EP/CPS/Co has delivered the best inhibition effect for smoke among all samples. The incorporation of CPS/Co leads to a remarkable reduction of PSPR by 33.3%, from 0.36 m^2^/s to 0.24 m^2^/s and a significant drop in TSR by 21.3%, from 2501 m^2^/m^2^ to 1970 m^2^/m^2^. In addition, thanks to the catalysis effect of Co for charring formation, compared to neat EP, the 3 wt.% addition of CPS/Co into EP increased char residue most, from 9.6% to 14.8%. The results of PHRR, THR, MARHE, TSR and residue suggest that CPS/Co has the best flame retardancy efficiency among all the samples.

### 3.4. Flame Retardant Mechanism

According to the TGA and cone test results above, with 3 wt.% addition of CPS (700) and CPS/Co, the fire safety of EP has been enhanced effectively, while the addition of CPS (180) brings little improvement. Following this, further study to explore flame retardant mechanisms herein focused on EP/CPS (700) and EP/CPS/Co.

It is widely recognised that the mechanism of the FRs can be divided into a gaseous phase and a condensed phase. To investigate the mechanisms, correspondingly, XPS, SEM, and TG-FTIR were employed to analyse char residue after the cone calorimeter test and the product gas during the thermal degradation process.

**Figure 6:**
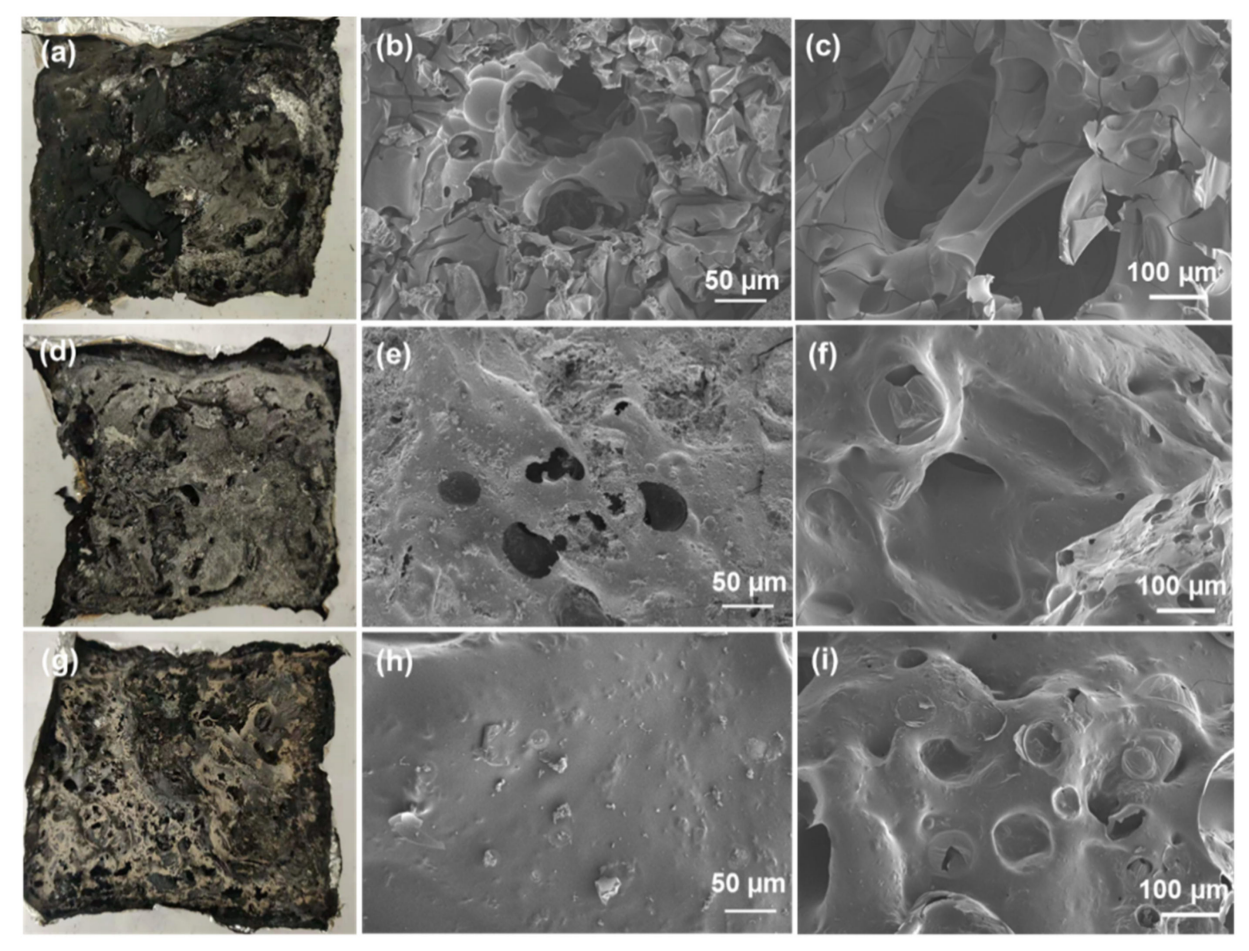
Digital photos of residues and SEM images of interior and exterior char residue for: (**a**–**c**) EP, (**d**–**f**) EP/CPS (700) and (**g**–**i**) EP/CPS/Co.

Figure 6 illustrates the SEM interior and exterior images of char residue of the specimens after the cone calorimeter test. Numerous cracks and wrinkles can be observed on the surface of neat EP, and holes of a large size are observed inside. For EP/CPS/Co, the char becomes denser than EP and EP/CPS (700), with a smaller porous structure. The compact char layer can suppress heat, volatile gas, and smoke infiltration, which leads to better fire safety behaviour and thermal stability.

**Figure 7:**
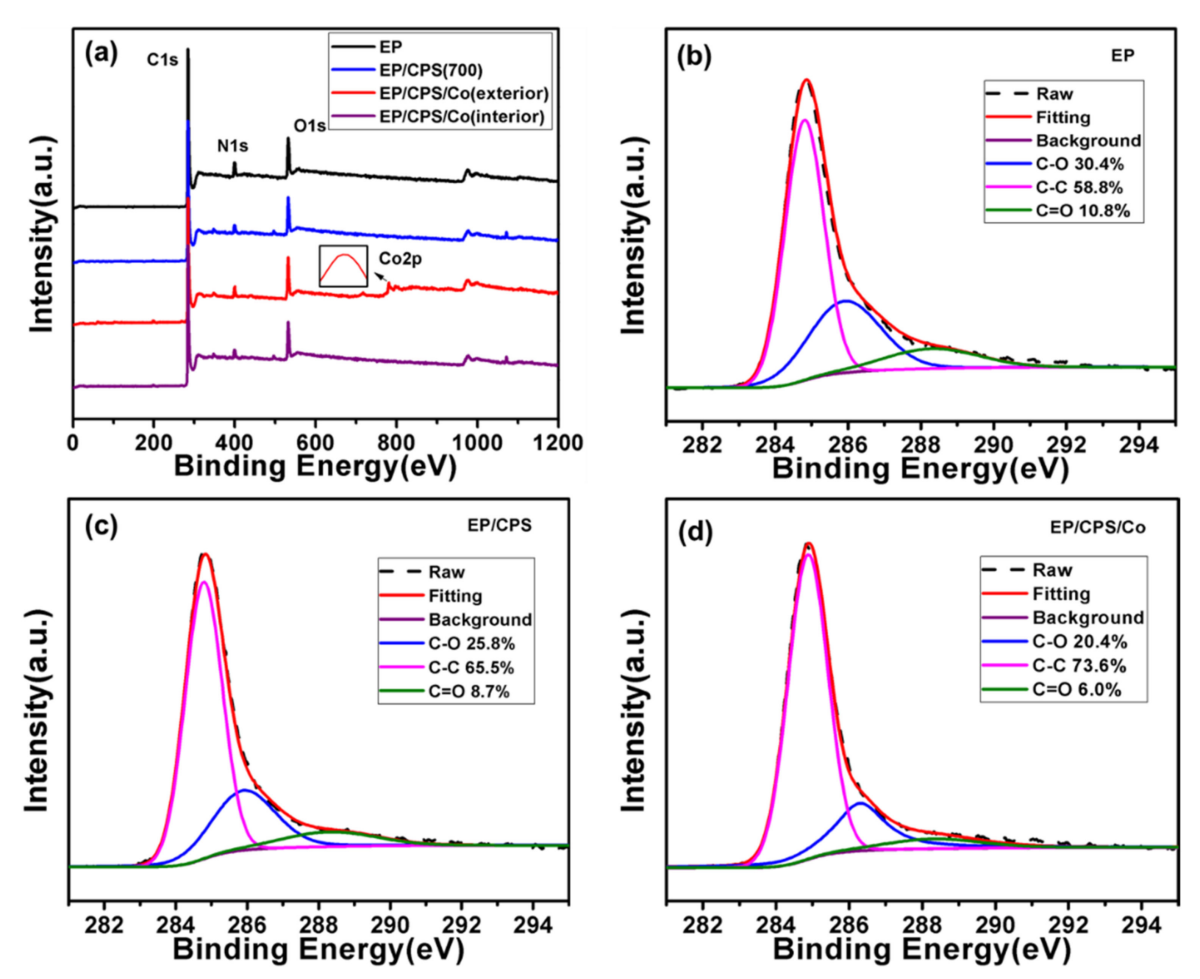
(**a**) XPS survey scans of interior char of EP and its composites and exterior char for EP/CPS/Co, (**b**–**d**) XPS C1s spectra of interior char of EP and its composites.

The XPS survey scans of the char residue are shown in Figure 7a. The samples include the interior char of EP and its composite and exterior char of EP/CPS/Co. It can be observed that the characteristic peak of the Cobalt element in the exterior char of EP/CPS/Co composite is stronger than the interior chars. To compare concentration changes of Co, mass percentage of Co in EP/CPS/Co composites and all the elements mass percentage in its char residue (interior and exterior) are listed in Table 4. The value of mass percentage of Co in EP/CPS/Co composites was calculated as 0.2%, according to the XPS result of Co mass percentage 7.5% for FR CPS/Co and 3 wt.% addition into EP. Table 4 shows that, after cone calorimeter test, Co concentration increased from 0.2% to 3.2% (interior) and 7.5% (exterior), indicating the degradation and vitalization of EP resin, and retention of Co in the condensed phase and, thus, its concentration grows. With more Co elements remaining in the exterior and its catalysis effect, the surface of the matrix can form a compact char layer during combustion, delaying the infiltration of heat, oxygen, and toxic gas.

Figure 7b–d present the high resolution of C1s XPS spectra of interior char. The characteristic C1s bands at 284.8 ev, 286.2 ev, 288.4 ev can be attributed to the C-H/C-C (aliphatic and aromatic species), C-O (hydroxy group and/or ether) and C=O, respectively [43]. In terms of the interior char’s aliphatic and aromatic species concentration, the relationship between EP composites is EP/CPS/Co > EP/CPS (700) > pure EP. This indicates that the char of EP/CPS/Co has the highest graphitisation degree among the samples [44]. The values of Cox/Ca (Cox denotes oxidised carbon, Ca denotes aliphatic and aromatic carbons) are calculated to compare the thermal-oxidative resistance of the matrix. The Cox/Ca values of EP/CPS/Co and EP/CPS are 0.36 and 0.53, respectively, which are much lower than pristine EP (0.7).

**Figure 8:**
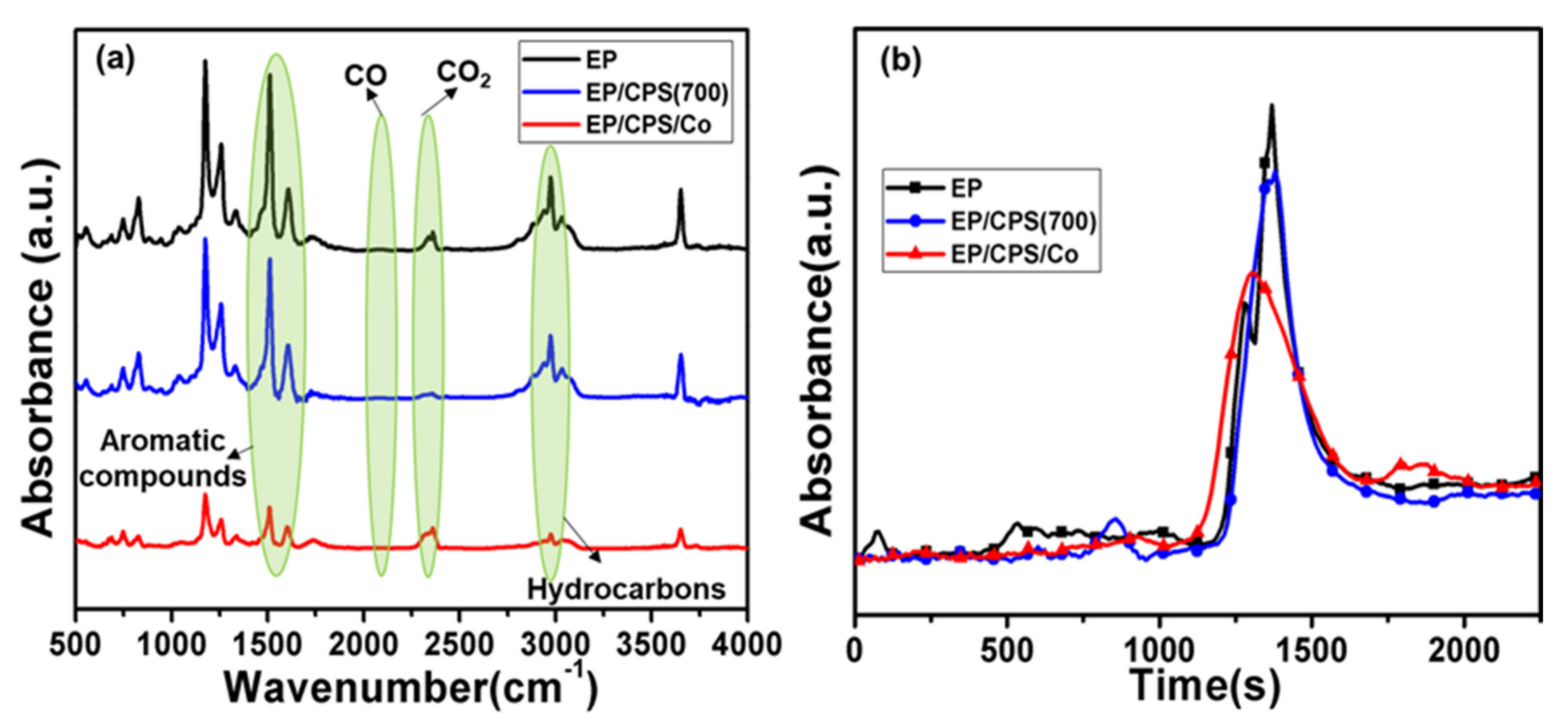
(**a**) FTIR spectrum of pyrolysis products at the maximum decomposition rate, (**b**) absorbance of decomposition products versus time (Gram-Schmidt).

To further explore the flame retardancy mechanisms, the gaseous phase was investigated by TG-FTIR, which is a semi quantitative analysis. The FTIR spectra of pyrolysis products of pure EP, EP/CPS (700) and EP/CPS/Co are revealed in Figure 8a. The intensity of absorbance is a relative value, and all the samples from the same batch were compared with pure EP. Some evolved gases are identified by the characteristic, unambiguous FTIR signals, including aromatic compounds (1407, 1510, 1607 cm^−1^), CO (2190 cm^−1^), CO_2_ (2360 cm^−1^), C-H groups for acetone, ethers, allyl alcohol and different kinds of hydrocarbons (2800–3100 cm^−1^) [45,46]. The Gram-Schmidt curves of EP and its composites are shown in Figure 8b. It shows that the absorbance of pyrolysis products from EP/CPS/Co is much lower than pristine EP, followed by EP/CPS (700).

**Figure 9:**
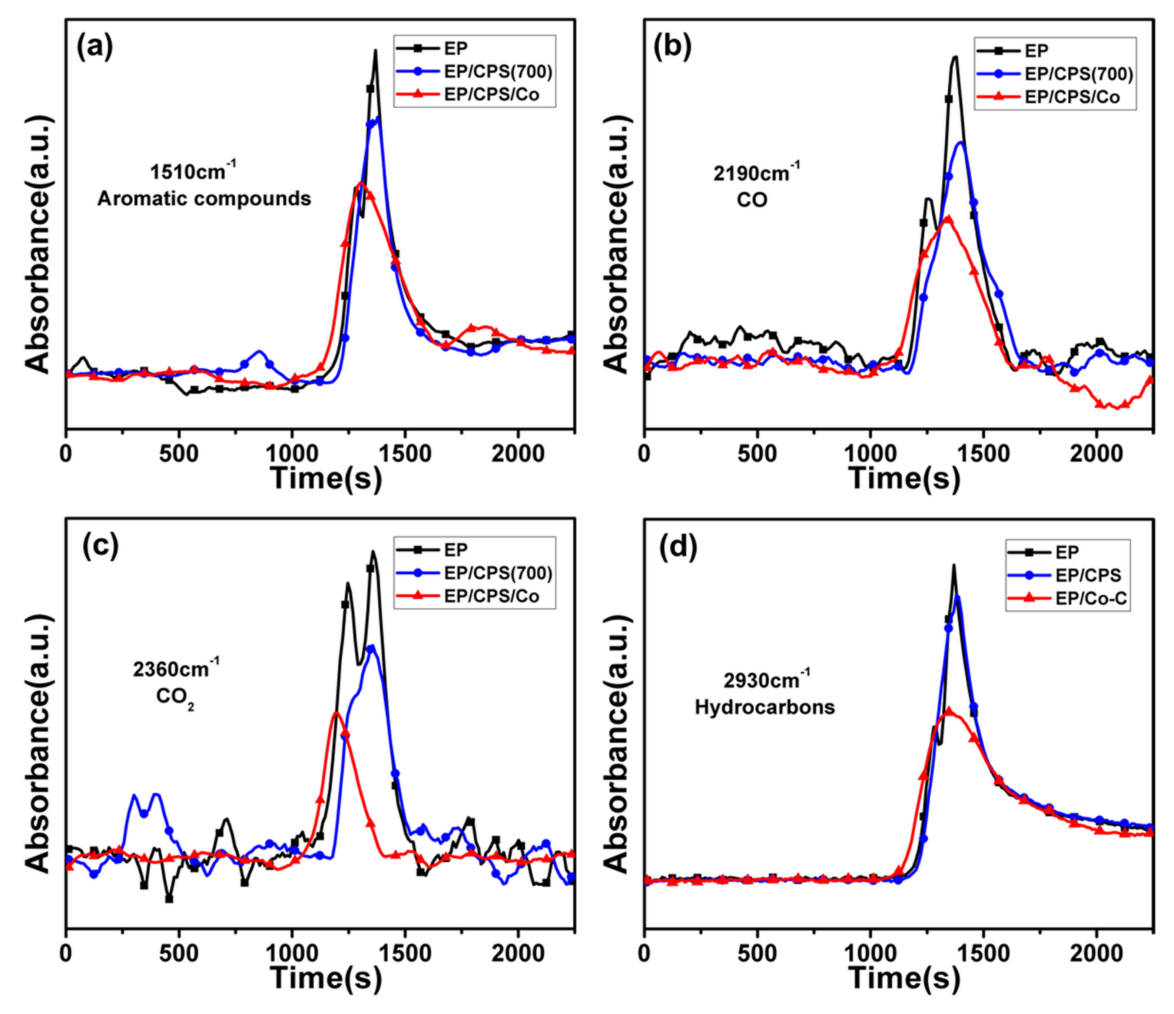
(**a**–**d**) Absorbance of volatile gas versus time for EP and its composites.

To analyse the effect of CPS (700) and CPS/Co on gases products (aromatic compounds, CO, CO_2_ and hydrocarbons), four main kinds of volatiles [47,48] from EP and its composites are further studied and presented in Figure 9a–d. Compared to EP, the absorbance of the pyrolysis products from EP/CPS (700) has slightly decreased. This is owed to the 2D structure of carbon derived from peanut shells with acceptable thermal stability, which functions as a physical barrier to delay the permeation of gaseous product and heat in the initial stage of decomposition (under 400 °C). The incorporation of Cobalt encourages a slightly earlier release of gas products, but a significantly lower peak intensity. Gaseous products are efficiently inhibited due to the physical barrier effect: the 2D structure of carbon from PS with high graphitization degree and catalysis effect of Cobalt for dense char formation. In addition, combustible gases such as hydrocarbons and aromatic compounds are simultaneously suppressed, which leads to the reduction of fuel for flame and smoke formation [49].

**Scheme 2:**
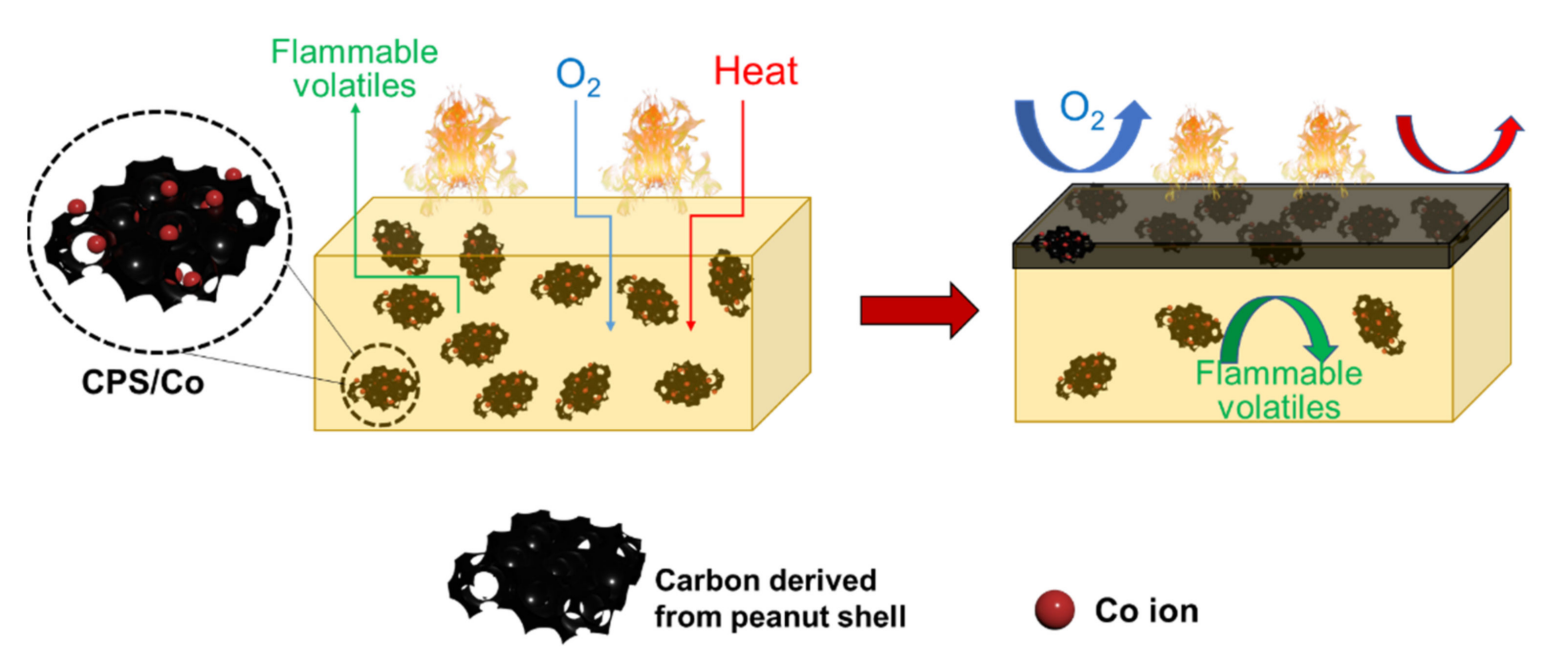
Flame retardancy mechanism of CPS/Co in EP composite.

In summary, the improvement in the fire performance of EP by adding CPS/Co can be mainly attributed to the synergistic effect from carbon derived from peanut shells and nano cobalt. Scheme 2 illustrates the flame retardancy mechanism. At the beginning of the condensed phase, the well-dispersed 2D carbon with a stable backbone can postpone the permeation of combustible gas, oxygen and radiation to some extent. At the same time, during combustion, the concentration of Cobalt in the matrix is increased and more Cobalt is retained on the surface. With the catalysis effect from transition metal Cobalt, both the graphitisation degree and char morphology are significantly improved in forming a compact and thermal stable char layer. It can shield the underlying matrix from feedback heat and oxygen, as well as flammable volatiles.

## 4. Conclusions

In this work, flame retardant fillers based on carbon derived from peanut shells were prepared through three facile methods, marked as CPS (180), CPS (700) and CPS/Co, to improve the fire safety performance of EP. The products were characterised by XRD, SEM and Raman. The layer structure derived from peanut shells can be observed clearly by SEM. After grinding, all the samples were reduced to small pieces of nanoplates with 2D structures. The Raman result shows that due to the catalysis effect of Cobalt, CPS/Co shows the highest graphitisation degree among the samples, which can provide more effective barriers against heat and gaseous products diffusion. As expected, TGA and cone calorimeter test results displayed that EP/CPS/Co offers the best fire performance. Only the addition of 3 wt% CPS/Co can lead to a dramatic reduction of pHRR and PSPR by 37.9% and 33.3%, respectively. Furthermore, the flame retardancy mechanism of CPS/Co was explored in terms of synergistic physical effect, which is attributed to the shield effect from the 2D structure of stable carbon and the catalysis effect of Cobalt for a dense char formation.

## Figures and Tables

**Table 1:** TGA data of EP and its composites.

Samples	Under Nitrogen		
	T_−5%_ (°C)	T_max_ (°C); %/min	Char Residue at 700 °C (%)
EP	372.92	391.7; 1.68	15.69
EP/CPS (180)	364.83	387.4; 1.65	15.25
EP/CPS (700)	375.03	390.3; 1.54	15.73
EP/CPS/Co	355.93	380.2; 1.42	18.31

**Table 2:** Cone calorimetry data of EP and its composites.

Samples	TTI (s)	pHRR (kw/m^2^)	THR (MJ/m^2^)	PSPR (m^2^/s)	TSR (m^2^/m^2^)	MARHE (kw/m^2^)	Residue (%)
EP	75 ± 1	1313 ± 32	102 ± 2	0.36 ± 0.02	2501 ± 45	471 ± 11	9.6 ± 0.3
EP/CPS (180)	73 ± 3	1180 ± 21	100 ± 3	0.31 ± 0.01	2444 ± 29	462 ± 7	8.2 ± 0.2
EP/CPS (700)	75 ± 1	1084 ± 25	89 ± 2	0.31 ± 0.02	2334 ± 37	455 ± 15	9.9 ± 0.2
EP/CPS/Co	77 ± 2	816 ± 29	82 ± 2	0.24 ± 0.01	1970 ± 17	426 ± 5	14.8 ± 0.3

**Table 3:** Percentage reductions (compared to neat EP) of key parameters for EP composites.

Samples	pHRR (%)	THR (%)	PSPR (%)	TSR (%)
EP	0	0	0	0
EP/CPS (180)	10.1	2.0	13.9	2.3
EP/CPS (700)	17.4	12.7	13.9	6.7
EP/CPS/Co	37.9	19.6	30.7	21.2

**Table 4:** Element analysis of EP/CPS/Co composite and its char residue (interior and exterior).

Samples	Mass Percentage (%)			
	C	O	N	Co
EP/CPS/Co	-	-	-	0.2
EP/CPS/Co char residue (interior)	82.9	10.1	3.7	3.2
EP/CPS/Co char residue (exterior)	74.6	13.4	4.5	7.5

## Data Availability

The data presented in this study are available in Supplementary Material.

## Supplementary Materials

The following are available online, Figure S1: Scanning electron microscopy images and corresponding elemental mapping images of CPS/Co: C:(green), O(purple) and Co(red), Figure S2: Scanning electron microscopy images of cross-sections of (a) EP, (b) EP/CPS (180), (c) EP/CPS (700) and (d) EP/CPS/Co, Figure S3: Raman spectra of the flame retardant CPS (180).

## References

[1] Rakotomalala M., Wagner S., Döring M. (2010). Recent developments in halogen free flame retardants for epoxy resins for electrical and electronic applications. Materials.

[2] Weil E.D., Levchik S. (2004). A Review of Current Flame Retardant Systems for Epoxy Resins. J. Fire Sci..

[3] Spontón M., Mercado L.A., Ronda J.C., Galià M., Cádiz V. (2008). Preparation, thermal properties and flame retardancy of phosphorus- and silicon-containing epoxy resins. Polym. Degrad. Stab..

[4] Liu Q., Wang D., Li Z., Li Z., Peng X., Liu C., Zhang Y., Zheng P. (2020). Recent developments in the flame-retardant system of epoxy resin. Materials.

[5] Dasari A., Yu Z.Z., Cai G.P., Mai Y.W. (2013). Recent developments in the fire retardancy of polymeric materials. Prog. Polym. Sci..

[6] Laoutid F., Bonnaud L., Alexandre M., Lopez-Cuesta J.M., Dubois P. (2009). New prospects in flame retardant polymer materials: From fundamentals to nanocomposites. Mater. Sci. Eng. R Rep..

[7] Wu C.S., Liu Y.L., Chiu Y.S. (2002). Epoxy resins possessing flame retardant elements from silicon incorporated epoxy compounds cured with phosphorus or nitrogen containing curing agents. Polymers.

[8] Huang G., Gao J., Wang X., Liang H., Ge C. (2012). How can graphene reduce the flammability of polymer nanocomposites?. Mater. Lett..

[9] Lee Y.R., Kim S.C., Lee H., Jeong H.M., Raghu A.V., Reddy K.R., Kim B.K. (2011). Graphite oxides as effective fire retardants of epoxy resin. Macromol. Res..

[10] Elbasuney S. (2015). Surface engineering of layered double hydroxide (LDH) nanoparticles for polymer flame retardancy. Powder Technol..

[11] Xu W., Li A., Liu Y., Chen R., Li W. (2018). CuMoO4@hexagonal boron nitride hybrid: An ecofriendly flame retardant for polyurethane elastomer. J. Mater. Sci..

[12] Pan Y., Wang W., Pan H., Zhan J., Hu Y. (2016). Fabrication of montmorillonite and titanate nanotube based coatings: Via layer-by-layer self-assembly method to enhance the thermal stability, flame retardancy and ultraviolet protection of polyethylene terephthalate (PET) fabric. RSC Adv..

[13] Cai W., Hong N., Feng X., Zeng W., Shi Y., Zhang Y., Wang B., Hu Y. (2017). A facile strategy to simultaneously exfoliate and functionalize boron nitride nanosheets via Lewis acid-base interaction. Chem. Eng. J..

[14] Madakbaş S., Çakmakçi E., Kahraman M.V. (2013). Preparation and thermal properties of polyacrylonitrile/hexagonal boron nitride composites. Thermochim. Acta.

[15] Wang X., Kalali E.N., Wang D.-Y. (2016). Two-Dimensional Inorganic Nanomaterials: A Solution to Flame Retardant Polymers. Nano Adv..

[16] Yue X., Li C., Ni Y., Xu Y., Wang J. (2019). Flame retardant nanocomposites based on 2D layered nanomaterials: A review. J. Mater. Sci..

[17] Das O., Sarmah A.K., Bhattacharyya D. (2016). Biocomposites from waste derived biochars: Mechanical, thermal, chemical, and morphological properties. Waste Manag..

[18] Babu K., Rendén G., Mensah R.A., Kim N.K., Jiang L., Xu Q., Restás Á., Neisiany R.E., Hedenqvist M.S., Försth M. (2020). A review on the flammability properties of carbon-based polymeric composites: State-of-the-art and future trends. Polymers.

[19] Nizamuddin S., Jadhav A., Qureshi S.S., Baloch H.A., Siddiqui M.T.H., Mubarak N.M., Griffin G., Madapusi S., Tanksale A., Ahamed M.I. (2019). Synthesis and characterization of polylactide/rice husk hydrochar composite. Sci. Rep..

[20] Matta S., Bartoli M., Frache A., Malucelli G. (2021). Investigation of different types of biochar on the thermal stability and fire retardance of ethylene-vinyl acetate copolymers. Polymers.

[21] Omega A.C.S. (2021). Intumescent-grafted bamboo charcoal: A natural nontoxic fire-retardant filler for polylactic acid (PLA) composites. ACS Omega.

[22] Wu M.F., Hsiao C.H., Lee C.Y., Tai N.H. (2020). Flexible Supercapacitors Prepared Using the Peanut-Shell-Based Carbon. ACS Omega.

[23] Ding J., Wang H., Li Z., Cui K., Karpuzov D., Tan X., Kohandehghan A., Mitlin D. (2015). Peanut shell hybrid sodium ion capacitor with extreme energy-power rivals lithium ion capacitors. Energy Environ. Sci..

[24] Wang H., Yu W., Shi J., Mao N., Chen S., Liu W. (2016). Biomass derived hierarchical porous carbons as high-performance anodes for sodium-ion batteries. Electrochim. Acta.

[25] Zheng Y., Lu Y., Zhou K. (2019). A novel exploration of metal–organic frameworks in flame-retardant epoxy composites. J. Therm. Anal. Calorim..

[26] Wu N., Yang R. (2011). Effects of metal oxides on intumescent flame-retardant polypropylene. Polym. Adv. Technol..

[27] Morgan A.B. (2010). Chapter 19 A Review of Transition Metal-Based Flame Retardants: Transition Metal Oxide/Salts, and Complexes the Periodic Table of Flame Retardants. Transition.

[28] Wang B., Zhou K., Jiang S., Hu Y., Gui Z. (2014). The application of transition metal molybdates (AMoO4, A = Co, Ni, Cu) as additives in acrylonitrile-butadiene-styrene with improved flame retardant and smoke suppression properties. Polym. Adv. Technol..

[29] Wang H., Qiao H., Guo J., Sun J., Li H., Zhang S., Gu X. (2020). Preparation of cobalt-based metal organic framework and its application as synergistic flame retardant in thermoplastic polyurethane (TPU). Compos. Part B Eng..

[30] López-Romero S., Chávez-Ramírez J. (2007). Synthesis of TiC thin films by CVD from toluene and titanium tetrachloride with nickel as catalyst. Matéria.

[31] Qiu T., Yang J.G., Bai X.J., Wang Y.L. (2019). The preparation of synthetic graphite materials with hierarchical pores from lignite by one-step impregnation and their characterization as dye absorbents. RSC Adv..

[32] Sinkó K., Szabó G., Zrínyi M. (2011). Liquid-phase synthesis of cobalt oxide nanoparticles. J. Nanosci. Nanotechnol..

[33] Dang N.M., Zhao W.W., Yusa S.I., Noguchi H., Nakashima K. (2015). Cobalt oxide hollow nanoparticles as synthesized by templating a tri-block copolymer micelle with a core-shell-corona structure: A promising anode material for lithium ion batteries. New J. Chem..

[34] Ansari S.M., Bhor R.D., Pai K.R., Sen D., Mazumder S., Ghosh K., Kolekar Y.D., Ramana C.V. (2017). Cobalt nanoparticles for biomedical applications: Facile synthesis, physiochemical characterization, cytotoxicity behavior and biocompatibility. Appl. Surf. Sci..

[35] Marton M., Vojs M., Zdravecká E., Himmerlich M., Haensel T., Krischok S., Kotlár M., Michniak P., Veselý M., Redhammer R. (2013). Raman spectroscopy of amorphous carbon prepared by pulsed arc discharge in various gas mixtures. J. Spectrosc..

[36] Pardanaud C., Martin C., Roubin P., Giacometti G., Hopf C., Schwarz-Selinger T., Jacob W. (2013). Raman spectroscopy investigation of the H content of heated hard amorphous carbon layers. Diam. Relat. Mater..

[37] Carosio F., Maddalena L., Gomez J., Saracco G., Fina A. (2018). Graphene Oxide Exoskeleton to Produce Self-Extinguishing, Nonignitable, and Flame Resistant Flexible Foams: A Mechanically Tough Alternative to Inorganic Aerogels. Adv. Mater. Interfaces.

[38] Yu B., Xing W., Guo W., Qiu S., Wang X., Lo S., Hu Y. (2016). Thermal exfoliation of hexagonal boron nitride for effective enhancements on thermal stability, flame retardancy and smoke suppression of epoxy resin nanocomposites: Via sol-gel process. J. Mater. Chem. A.

[39] Sadezky A., Muckenhuber H., Grothe H., Niessner R., Pöschl U. (2005). Raman microspectroscopy of soot and related carbonaceous materials: Spectral analysis and structural information. Carbon N. Y..

[40] Zhou K., Liu J., Shi Y., Jiang S., Wang D., Hu Y., Gui Z. (2015). MoS2 nanolayers grown on carbon nanotubes: An advanced reinforcement for epoxy composites. ACS Appl. Mater. Interfaces.

[41] Lin B., Yuen A.C.Y., Chen T.B.Y., Yu B., Yang W., Zhang J., Yao Y., Wu S., Wang C.H., Yeoh G.H. (2021). Experimental and numerical perspective on the fire performance of MXene/Chitosan/Phytic acid coated flexible polyurethane foam. Sci. Rep..

[42] Salasinska K., Celiński M., Mizera K., Kozikowski P., Leszczyński M.K., Gajek A. (2020). Synergistic effect between histidine phosphate complex and hazelnut shell for flammability reduction of low-smoke emission epoxy resin. Polym. Degrad. Stab..

[43] Wang W., Pan H., Shi Y., Pan Y., Yang W., Liew K.M., Song L., Hu Y. (2016). Fabrication of LDH nanosheets on β-FeOOH rods and applications for improving the fire safety of epoxy resin. Compos. Part A Appl. Sci. Manuf..

[44] Bourbigot S., Le Bras M., Delobel R., Gengembre L. (1997). XPS study of an intumescent coating. Appl. Surf. Sci..

[45] Wang X., Zhou S., Xing W., Yu B., Feng X., Song L., Hu Y. (2013). Self-assembly of Ni-Fe layered double hydroxide/graphene hybrids for reducing fire hazard in epoxy composites. J. Mater. Chem. A.

[46] Wang W., Kan Y., Yu B., Pan Y., Liew K.M., Song L., Hu Y. (2017). Synthesis of MnO_2_ nanoparticles with different morphologies and application for improving the fire safety of epoxy. Compos. Part A Appl. Sci. Manuf..

[47] Qiu S., Zou B., Zhang T., Ren X., Yu B., Zhou Y., Kan Y., Hu Y. (2020). Integrated effect of NH_2_-functionalized/triazine based covalent organic framework black phosphorus on reducing fire hazards of epoxy nanocomposites. Chem. Eng. J..

[48] Qiu S., Zhou Y., Zhou X., Zhang T., Wang C., Yuen R.K.K., Hu W., Hu Y. (2019). Air-Stable Polyphosphazene-Functionalized Few-Layer Black Phosphorene for Flame Retardancy of Epoxy Resins. Small.

[49] Wang D., Zhang Q., Zhou K., Yang W., Hu Y., Gong X. (2014). The influence of manganese-cobalt oxide/graphene on reducing fire hazards of poly(butylene terephthalate). J. Hazard. Mater..

