# Supplementary material for: Peanut Shell Derived Carbon Combined with Nano Cobalt: An Effective Flame Retardant for Epoxy Resin
Source: Molecules. 2021 Nov 3;26(21):6662. doi: 10.3390/molecules26216662 (PMC8586977; doi:10.3390/molecules26216662)
Supplement: Supplementary file 1 [file molecules-26-06662-s001.zip › molecules-1403583-supplementary.pdf]

Supporting information for

Peanut shell derived carbon combined with nano Cobalt: an effective flame retardant for epoxy resin

Jing Liang [1,†], Wenhao Yang [2,†], Anthony Chun Yin Yuen [1,*], Hu Long [3], Shuilai Qiu [2,*], Ivan Miguel De Cachinho Cordeiro [1], Wei Wang [1], Timothy Bo Yuan Chen [1], Yuan Hu [2], Guan Heng Yeoh [1]

[1] School of Mechanical and Manufacturing Engineering, University of New South Wales, Sydney, NSW 2052, Australia;

[2] State Key Laboratory of Fire Science, University of Science and Technology of China, 96 Jinzhai Road, Hefei, Anhui 230026, PR China;

[3] School of Mechanical Science and Engineering, Huazhong University of Science and Technology, Wuhan 430074, China;

* Correspondence:
c.y.yuen@unsw.edu.au; Tel.: +61 2 9385 5697; fax: +61 2 9663 1222. (A.Y.)
shuilai@ustc.edu.cn; Tel & fax: +86 551 63602353. (S.Q.)

† These authors contributed equally to this work

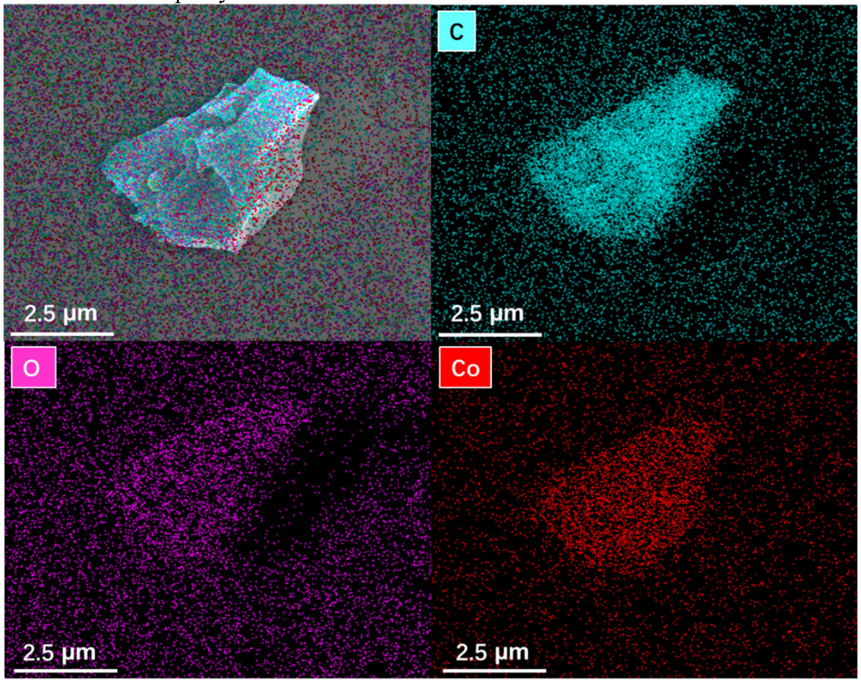

Figure S1. Scanning electron microscopy images and corresponding elemental mapping images of CPS/Co: C:(green), O(purple) and Co(red)

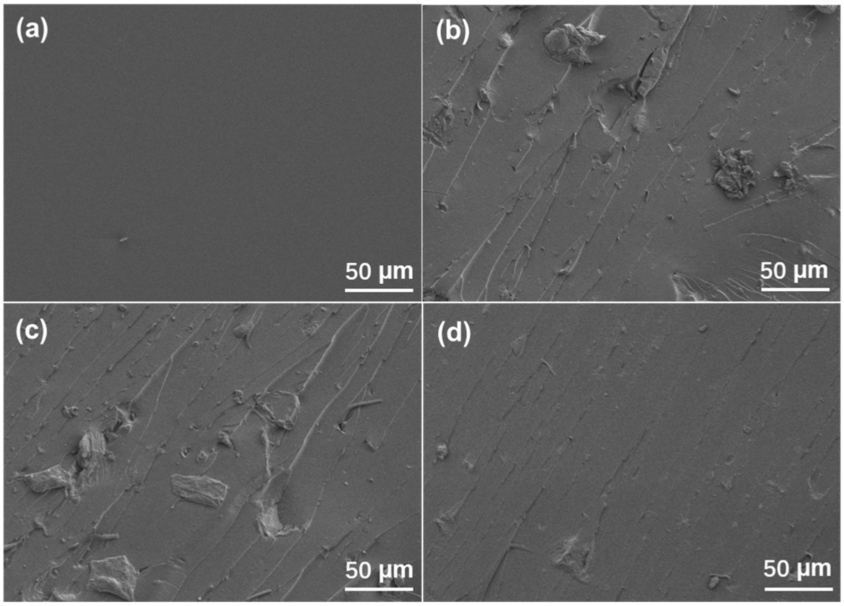

Figure S2. Scanning electron microscopy images of cross-sections of
(a) EP, (b) EP/CPS (180), (c) EP/CPS (700) and (d) EP/CPS/Co

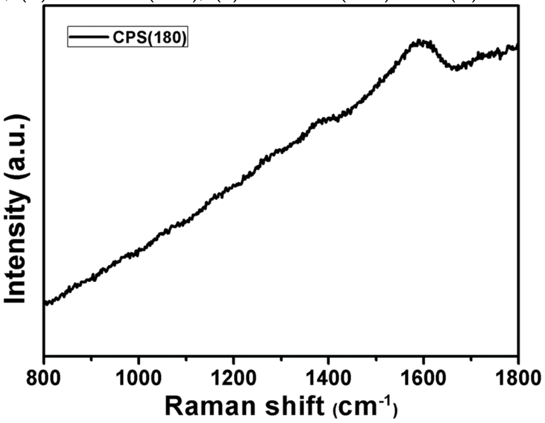

Figure S3. Raman spectra of the flame retardant CPS (180)
